# A Chatbot Versus Physicians to Provide Information for Patients With Breast Cancer: Blind, Randomized Controlled Noninferiority Trial

**DOI:** 10.2196/15787

**Published:** 2019-11-27

**Authors:** Jean-Emmanuel Bibault, Benjamin Chaix, Arthur Guillemassé, Sophie Cousin, Alexandre Escande, Morgane Perrin, Arthur Pienkowski, Guillaume Delamon, Pierre Nectoux, Benoît Brouard

**Affiliations:** 1 Department of Radiation Oncology Hôpital Européen Georges Pompidou, AP-HP Paris France; 2 ENT Department Hôpital Gui de Chauliac Université Montpellier 1 Montpellier France; 3 Wefight Institut du Cerveau et de la Moelle épinière Hôpital Pitié-Salpêtrière Paris France; 4 Department of Medical Oncology Institut Bergonié Bordeaux France; 5 Department of Radiation Oncology, Centre Oscar Lambret Lille France; 6 Department of Gynecological Oncologic Surgery Gustave Roussy Cancer Campus Villejuif France

**Keywords:** chatbot, clinical trial, cancer

## Abstract

**Background:**

The data regarding the use of conversational agents in oncology are scarce.

**Objective:**

The aim of this study was to verify whether an artificial conversational agent was able to provide answers to patients with breast cancer with a level of satisfaction similar to the answers given by a group of physicians.

**Methods:**

This study is a blind, noninferiority randomized controlled trial that compared the information given by the chatbot, Vik, with that given by a multidisciplinary group of physicians to patients with breast cancer. Patients were women with breast cancer in treatment or in remission. The European Organisation for Research and Treatment of Cancer Quality of Life Group information questionnaire (EORTC QLQ-INFO25) was adapted and used to compare the quality of the information provided to patients by the physician or the chatbot. The primary outcome was to show that the answers given by the Vik chatbot to common questions asked by patients with breast cancer about their therapy management are at least as satisfying as answers given by a multidisciplinary medical committee by comparing the success rate in each group (defined by a score above 3). The secondary objective was to compare the average scores obtained by the chatbot and physicians for each INFO25 item.

**Results:**

A total of 142 patients were included and randomized into two groups of 71. They were all female with a mean age of 42 years (SD 19). The success rates (as defined by a score >3) was 69% (49/71) in the chatbot group versus 64% (46/71) in the physicians group. The binomial test showed the noninferiority (*P*<.001) of the chatbot’s answers.

**Conclusions:**

This is the first study that assessed an artificial conversational agent used to inform patients with cancer. The EORTC INFO25 scores from the chatbot were found to be noninferior to the scores of the physicians. Artificial conversational agents may save patients with minor health concerns from a visit to the doctor. This could allow clinicians to spend more time to treat patients who need a consultation the most.

**Trial Registration:**

Clinicaltrials.gov NCT03556813, https://tinyurl.com/rgtlehq

## Introduction

### Background

Chatbots can imitate human conversation by using a field of artificial intelligence (AI) known as natural language processing. Chatbots are now widely used in several forms as voice-based agents, such as Siri (Apple), Google Now (Google), Alexa (Amazon), or Cortana (Microsoft). Text-based chatbots are available as Messenger (Facebook) agents or as stand-alone mobile or Web apps. They provide information and create a dynamic interaction between the agent and the user, without human back-end intervention. The concept of an artificial conversational agent dates back to 1950, when Alan Turing envisioned a future where a computer would be able to express itself with a level of sophistication that would render it indistinguishable from humans [1].

In health care, the first example of a computer program used as a conversational agent was Joseph Weizenbaum’s ELIZA, a program that mimicked a Rogerian psychotherapist and that was able to rephrase the patient’s sentences as questions and provide prerecorded answers [2]. In 1991, Dr Sbaitso was created as an AI speech synthesis program for MS-DOS personal computers. In this software, Dr Sbaitso was designed as a psychologist, with very limited possibilities [3]. Four years later, the chatbot, Artificial Linguistic Internet Computer Entity, was created to include 40,000 knowledge categories and was awarded the Loebner Prize thrice [4]. In 2001, SmarterChild was made available as a bot distributed across SMS networks and is now considered as a precursor to Apple’s Siri, which was released on iPhones in 2010. Patients can now use chatbots to check for symptoms and to monitor their health, but the relevance and validity of chatbots have rarely been assessed [5-7].

### Objective

Wefight designed a chatbot named Vik for patients with breast cancer and their relatives via personalized text messages. Vik provides information about breast cancer and its epidemiology, treatments, side effects, and quality of life improvement strategies (sport, fertility, sexuality, and diet). More practical information, such as reimbursement and patients’ rights, is also available. Chaix et al [8] showed that it was possible to obtain support through a chatbot as Vik improved the medication adherence rate of patients with breast cancer. Vik is available for free on the Web, on any mobile phones, iOS (Apple) or Android (Google), or on Messenger (Facebook).

This study is a blind, noninferiority randomized controlled trial that compared the information given by the Vik chatbot versus that given by a multidisciplinary group of physicians (medical, radiation, and surgical oncology) to patients with breast cancer (NCT03556813). The EORTC QLQ-INFO25 questionnaire, which was validated to assess information of patients with cancer [9], was adapted and used to compare the quality of the information provided to the 2 groups of patients by the physician or the chatbot.

## Methods

### Study Design and Participants

The study was a blind, noninterventional, noninferiority randomized study, without any risk or burden. It was conducted in France in November and December 2018.

**Figure 1:**
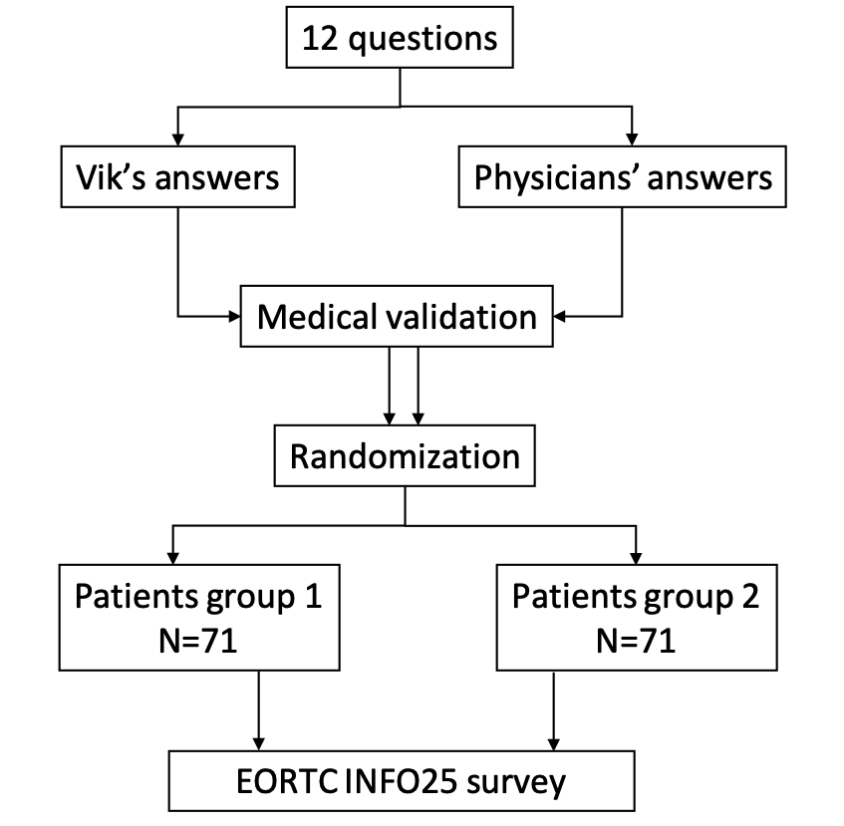
Flow diagram. EORTC QLQ-INFO25: European Organisation for Research and Treatment of Cancer.

The authors selected the 12 most frequently asked questions about breast cancer from Vik's database (Multimedia Appendix 1). These questions were then asked both to the Vik chatbot and to a multidisciplinary medical committee (oncologist surgeon, medical oncologist, and oncologist radiotherapist; Figure 1). The second independent multidisciplinary group of physicians ensured that each group’s answers did not provide inaccurate information. Institutional affiliations of the coordinating team were not displayed.

Patients were recruited with the help of a French breast cancer patients association (Mon Réseau Cancer du Sein). They were filtered for eligibility based on the inclusion criteria (age >18 years, female, subjects with breast cancer in treatment or remission, nonopposition, and internet literacy). Participants were compensated for their time. This study was approved by an ethics committee independently selected by the French Ministry of Health (N° ID RCB: 2018-A01365-50) and registered in the ClinicalTrials.gov database (NCT03556813). The data collected were anonymized and then hosted by Wefight on a server compliant with health care data storage requirements. Consent was collected online before the start of the study. In accordance with the French and European laws on information technology and civil liberties (Commission Nationale Informatique et Libertés, Règlement Général pour la Protection des Données), users had a right of use at their disposal to verify its accuracy and, if necessary, to correct, complete, and update it. They also had a right to object to their use and a right to delete these data. General conditions of use were displayed and explained very clearly, and they must be accepted before using the questionnaire. No demographical data beyond age were asked or gathered to participate in the study.

### Chatbot Design

Wefight designed a chatbot named Vik to empower patients with cancer and their relatives via personalized text messages. Vik’s answers are very diverse, and patients can find all the relevant, quality-checked medical information they need. Vik’s architecture is composed of several technological parts allowing a fine analysis of the questions posed by the patients and an adapted treatment of the answer.

For a chatbot to be fully developed, both machine learning algorithms and natural language processing are required. To build a chatbot, there are 2 crucial components to be supervised: intent classification and entity recognition. To understand the users’ messages and send personalized answers, the conversation goes through 3 steps: the first step analyzes the sentence and identifies intents and entities by using machine learning. The second stage activates modules according to the intents and entities detected by the first stage, and the third stage aggregates the answers of all activated modules to build the answer sent to the user and saves the conversation on the user’s profile.

For the patient, the use of a chatbot is very simple. It is a classic chat on a conversation window. The patient asks a question by writing it on his or her keyboard, and the chatbot answers directly in simple and understandable language.

### Procedures

Patients were randomized (1:1) blindly and received either the responses of the Vik chatbot or the responses of the medical committee to the predefined 12 questions, as previously explained. Participants were shown each question in order, and blinded responses were directly delivered as Web-based text messages for each group. The full answer for each question from either Vik or experts was directly shown to the participants upon activation of each question by the participants. There was no actual conversation per question nor the necessity of natural language processing for each question. Patients were then asked to complete an adapted version of the EORTC QLQ-INFO25 questionnaire online, assessing the quality of medical information received based on each response. A total of 21 items of the EORTC QLQ-INFO25 questionnaire were included (Multimedia Appendix 1).

### Outcomes

The perceived quality of the answers was assessed using the QLQ-INFO25 questionnaire that uses a scale of satisfaction graded from 1 to 4.

The primary objective was to assess the overall perceived quality of the answers given by the Vik chatbot to common questions asked by patients with breast cancer about their therapy management compared with answers given by a multidisciplinary medical committee (oncologist surgeon, medical oncologist, and radiotherapist oncologist), by comparing proportions of success in the physicians’ and Vik’s group. The secondary objective was to compare the average scores obtained by the chatbot and by the physicians for each individual INFO25 item. Gradings for the 21 items were averaged to define an overall score for each patient, in each group. We defined success as a grade greater than or equal to 3. Descriptive statistics were used to summarize patient characteristics by treatment group.

### Statistical Analysis

This study used a randomized phase III design with an alpha of .05 and a beta of .2 with a noninferiority limit of 10%. The effect size was based on a published EORTC INFO25 validation study [9]. This noninferiority limit of 10% was chosen as an acceptable difference for patient satisfaction. In view of these assumptions, the trial required at least 142 patients randomly assigned to the 2 groups. A 1-sided binomial test using the method of Mietinen and Nurminen was performed to compare the difference between the proportions of success in the 2 groups for questions 1 to 19 and the noninferiority limit. Noninferiority was declared if the *P* value of the test is lower than .05. For each item, confidence interval of the difference between the proportions of success in the physicians’ group and Vik’s group was estimated using the Wald Z method. Noninferiority was declared when the upper limit of a 2-sided 90% CI, equivalent to a 1-sided 95% CI, did not exceed the noninferiority limit of 10%.

## Results

### Analysis Size

Between November and December 2018, we included a total of 142 patients, divided into 2 groups of 71. For each group, the numbers of participants who were randomly assigned received the intended treatment and were analyzed for the primary outcome. A single intervention was performed for this study. All participating patients finished the evaluation. They were all female with a mean age of 42 years (SD 19).

### Descriptive Analysis

Patients responded to the questionnaire in an average of 15 min (SD 4). The first group of 71 patients received the responses from Vik, and the second group received the responses from physicians. The average global rating was 2.86 (median 3, IQR 2-4). The success rates (as defined by a score >3) were 69% in the chatbot group versus 64% in the physicians group. Patients assessing physicians’ answers gave an average rating of 2.82, whereas patients assessing Vik’s answers gave an average rating of 2.89 (Multimedia Appendix 2).

A total of 62.0% of patients (88/142) would have liked to get even more information (65% [46/71]) in the physicians’ group and 59% ([42/71] in Vik's group), whereas only 4.2% (6/142) would have liked to get less. A total of 83.1% of patients (118/142) found answers helpful (82% [58/71] in the physicians’ group and 85% [60/71] in Vik’s group), and 81.0% (115/142) were satisfied with the amount of information they have received (77% [55/71] in physicians’ group and 85% [60/71] in Vik's group).

### Comparison of Patient Groups

#### Primary Objective

The difference between success rates in the physicians’ group and Vik’s group was –0.03 (95% CI -0.07 to 0.00). Furthermore, the binomial test showed a noninferiority (P<1e-14) between the perceived quality of the chatbot responses and that of the physicians, as assessed by EORTC INFO25.

#### Secondary Objective

**Figure 2:**
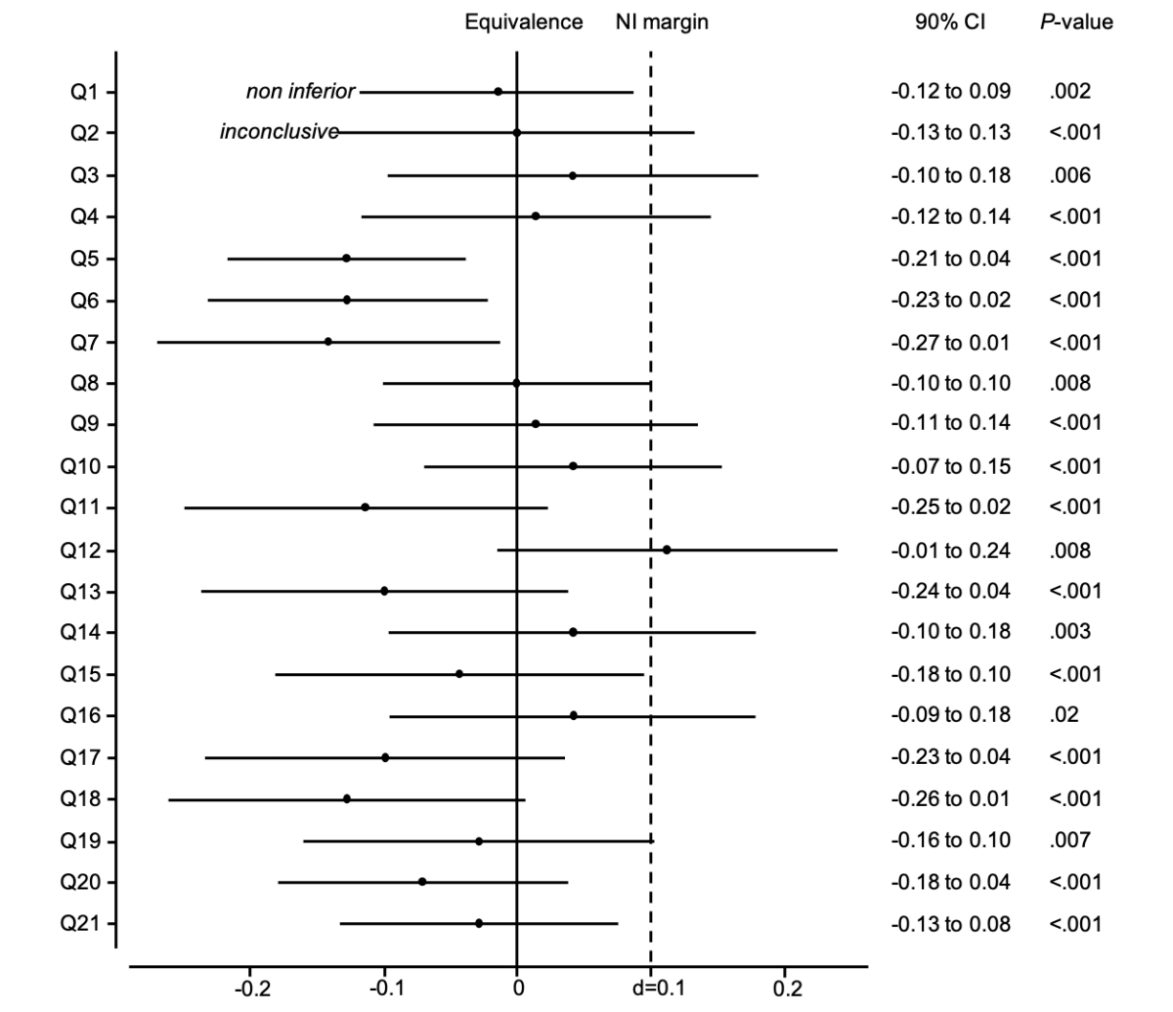
Noninferiority (NI) graph.

Both-sided 90% CI, equivalent to 1-sided 95% CI was computed for the difference between proportions of success in the physicians’ group and Vik’s group for each item (Multimedia Appendix 2). For 12 items of them, the noninferiority can be declared as the upper limit of the 95% CI did not exceed the 0.1 noninferiority limit (Figure 2). For the rest of them (9 items), the upper limit of the 95% CI crossed the 0.1 noninferiority limit. For these items, the noninferiority cannot be claimed. These items include questions 2 and 3 about breast cancer stages and causes, question 4 about whether or not the cancer is under control, 4 questions related to treatments (types, benefits, and side effects), and 2 questions related to care outside of the hospital.

## Discussion

### Principal Findings

This is the first study that rigorously assessed an artificial conversational agent used to inform patients with cancer, but the study has limitations: we did not evaluate the demographic features of the patients who answered the survey to remain in compliance with the European General Data Protection Regulation. Patients were recruited in our study through a patients association mailing list, which means that they could potentially be younger than the average population of patients with breast cancer, have more digital literacy skills, and be more open-minded toward digital tools, even if the 2 groups were blinded and did not know if they received the answers from the chatbot or from the group of physicians.

### Chatbot Assessment

A search on ClinicalTrials.gov currently returns only 4 trials evaluating chatbots in health care: in the United Kingdom, a nonrandomized trial is being performed by the National Health Service to compare the Babylon chatbot with the nonemergency 111 telephone number [10]. Patients interact with an automatic agent to describe their symptoms. Advices and information are given in return by the chatbot. The second trial, The Buddy Study (NCT02742740) evaluates an Embodied Conversational Agent (ECA) Oncology Trial Advisor for Cancer Trials that acts as an advisor to patients on chemotherapy regimens, promoting protocol adherence and retention, providing anticipatory guidance, and answering questions. The chatbot also serves as a conduit to capture information about complaints or adverse events. Usability metrics will include session time, satisfaction, and error rates. Subjects will be identified from among patients on chemotherapy regimens at the Boston Medical Center [11]. All subjects will be enrolled for 2 months and randomized to the chatbot group or control group. The primary outcome will be treatment protocol adherence, defined by the number of treatment visits attended/number of treatment visits scheduled. The secondary outcome will measure subject satisfaction, number of adverse events as reported through the ECA and directly to clinic by patient, time to detect and resolve adverse events as reported through the ECA and directly to clinic by patient, and adverse event false alarm rate as reported through ECA and directly to clinic by patient. The third study, the RAISE project (NCT01458002) [12], is designed to promote exercise and sun protection. The primary aims were to develop and assess the effectiveness of a tailored internet intervention on a national sample, to develop and assess the effectiveness of the internet intervention enhanced by a relational agent, and to determine if the intervention with the relational agent can outperform the regular tailored internet intervention. The study will include 3 groups (control, internet, and internet plus relational agent). A representative national sample of 1639 individuals at risk for both behaviors will be recruited.

Randomized studies demonstrating the superiority (or at least noninferiority) of chatbots, compared with an intervention performed by a physician, do not exist. However, if chatbots are to be safely used by a large number of patients, they must be evaluated like a medical device or even a drug. The consequences of a medical chatbot dysfunction could potentially have a significant negative impact, such as misdiagnosis, delayed diagnosis, inappropriate self-medication, or bad treatment adherence. Their use should not be promoted without conducting thorough investigations.

### Conclusions

The data regarding the use of conversational agents in health care in general and oncology in particular are limited, which is in sharp contrast with their potential benefits for the patients and the health care system. In this phase III, blind, noninferiority, randomized controlled trial, the EORTC INFO25 scores from the chatbot were found to be noninferior to the scores of the group of physicians. Conversational agents may save patients with minor health concerns from a visit to the doctor. This could allow clinicians to spend more time to treat patients who need a consultation at the most. Consultations for symptoms that do not require an actual consultation could be avoided, potentially saving a significant amount of money and resources. However, if the quality of these computer programs is not rigorously assessed, they could be unable to actually detect the difference between minor and major symptoms, without anyone knowing. Health chatbots will need to be used by many and have access to rich datasets to increase their knowledge of medical terms, symptoms, and treatments. These systems will not replace the physicians and should be considered as a resource to enhance the efficacy of health care interventions. If chatbots are consistently shown to be effective and safe, they could be prescribed like a drug to improve patient information, monitoring, or treatment adherence. Significant hurdles still exist in the widespread application of chatbots at this time, such as compliance with the Health Insurance Portability and Accountability Act.

## Appendix

Multimedia Appendix 1 Questions used and adapted version of the EORTC QLQ-INFO25 questionnaire.

Multimedia Appendix 2 Detailed grading of each EORTC INFO25 item in each group.

Multimedia Appendix 3 CONSORT-EHEALTH checklist (V 1.6.1).

## Abbreviations

AI: artificial intelligence
ECA: Embodied Conversational Agent
EORTC QLQ-INFO25: European Organisation for Research and Treatment of Cancer Quality of Life Group information questionnaire
