# Supplementary material for: A Chatbot Versus Physicians to Provide Information for Patients With Breast Cancer: Blind, Randomized Controlled Noninferiority Trial
Source: J Med Internet Res. 2019 Nov 27;21(11):e15787. doi: 10.2196/15787 (PMC6906616; doi:10.2196/15787)
Supplement: Multimedia Appendix 1 [file jmir_v21i11e15787_app1.docx]

MA 1. Questions used and adapted version of the EORTC QLQ-INFO25 questionnaire.

| Questions asked to Vik and physicians; information received on: | | Not at all | | A little | Quite a bit | Very much | |
| --- | --- | --- | --- | --- | --- | --- | --- |
| **How is breast cancer diagnosed?** | | | | | | | |
|  | 1. The diagnosis of your disease? | 1 | | 2 | 3 | 4 | |
| **What are the stages of cancer?** | | | | | | | |
|  | 2. The extent (spread) of your disease? | 1 | | 2 | 3 | 4 | |
| **What causes my cancer?** | | | | | | | |
|  | 3. The possible causes of your disease? | 1 | | 2 | 3 | 4 | |
| **Is my cancer under control?** | | | | | | | |
|  | 4. Whether the disease is under control? | 1 | | 2 | 3 | 4 | |
| **What are the purposes of the exams I will do? How do they proceed?** | | | | | | | |
|  | 5. The purpose of any medical tests you have had or may undergo? | 1 | | 2 | 3 | 4 | |
|  | 6. The procedures of the medical tests? | 1 | | 2 | 3 | 4 | |
|  | 7. The results of the medical tests you have already received? | 1 | | 2 | 3 | 4 | |
| **What are the treatments for breast cancer and their benefits?** | | | | | | | |
|  | 8. The medical treatment (chemotherapy, radiotherapy, surgery, or other treatment modality)? | 1 | | 2 | 3 | 4 | |
|  | 9. The expected benefit of the treatment? | 1 | | 2 | 3 | 4 | |
| **What are the side effects of chemotherapy or hormone therapy?** | | | | | | | |
|  | 10. The possible side-effects of your treatment? | 1 | | 2 | 3 | 4 | |
|  | 11. The effects of the treatment on social and family life? | 1 | | 2 | 3 | 4 | |
| **How will the treatments affect the symptoms of my cancer?** | | | | | | | |
|  | 12. The expected effects of the treatment on disease symptoms? | 1 | | 2 | 3 | 4 | |
| **What impact will the treatments have on my sex life?** | | | | | | | |
|  | 13. The effects of the treatment on sexual activity? | 1 | | 2 | 3 | 4 | |
| **What help can I get outside the hospital to manage my cancer at home?** | | | | | | | |
|  | 14. Additional help outside the hospital (eg, help with daily activities, self–help groups, and district nurses)? | 1 | | 2 | 3 | 4 | |
|  | 15. Aspects of managing your illness at home? | 1 | | 2 | 3 | 4 | |
|  | 16. Different places of care (hospitals/outpatient services/home)? | 1 | | 2 | 3 | 4 | |
| **What complementary or rehabilitative care can I do?** | | | | | | | |
|  | 17. Rehabilitation services (eg, physiotherapy and occupational therapy)? | 1 | | 2 | 3 | 4 | |
|  | 18. Things that you can do to help yourself get well (rest, contact with others)? | 1 | | 2 | 3 | 4 | |
| **How and who can help me psychologically?** | | | | | | | |
|  | 19. Possible professional psychological support? | 1 | | 2 | 3 | 4 | |
| **Global satisfaction** | | | | | | | |
|  | 20. Were you satisfied with the amount of information you received? | 1 | | 2 | 3 | 4 | |
|  | 21. Overall has the information you have received been helpful? | 1 | | 2 | 3 | 4 | |
|  | Do you wish to receive more information? | Yes | Yes | | No | | No |
|  | Do you wish that you had received less information? | Yes | Yes | | No | | No |
