# Supplementary material for: A Chatbot Versus Physicians to Provide Information for Patients With Breast Cancer: Blind, Randomized Controlled Noninferiority Trial
Source: J Med Internet Res. 2019 Nov 27;21(11):e15787. doi: 10.2196/15787 (PMC6906616; doi:10.2196/15787)
Supplement: Multimedia Appendix 2 [file jmir_v21i11e15787_app2.docx]

MA 2. Detailed grading of each EORTC INFO25 item in each group.

| Label | Mean (SD) | | | First quartile | | | Median | | | Third quartile | | | *P* value | 90% CI | P1 (Physician) | P2 (Vik) |
| --- | --- | --- | --- | --- | --- | --- | --- | --- | --- | --- | --- | --- | --- | --- | --- | --- |
|  | Physicians | Vik | Total | Physicians | Vik | Total | Physicians | Vik | Total | Physicians | Vik | Total |  |  |  |  |
|  | | | | | | | | | | | | | | | | |
| Q1 | 3.31 (0.75) | 3.17 (0.83) | 3.24 (0.79) | 3.00 | 3.00 | 3.00 | 3.00 | 3.00 | 3.00 | 4.00 | 4.00 | 4.00 | .002 | -0.12 to 0.09 | 0.83 | 0.85 |
| Q2 | 2.86 (0.88) | 2.87 (0.89) | 2.87 (0.89) | 2.00 | 2.00 | 2.00 | 3.00 | 3.00 | 3.00 | 4.00 | 4.00 | 4.00 | <.001 | -0.13 to 0.13 | 0.65 | 0.65 |
| Q3 | 2.65 (1.07) | 2.61 (0.93) | 2.63 (1.00) | 2.00 | 2.00 | 2.00 | 3.00 | 2.00 | 3.00 | 4.00 | 3.00 | 3.00 | .006 | -0.10 to 0.18 | 0.54 | 0.49 |
| Q4 | 2.83 (0.86) | 2.85 (0.82) | 2.84 (0.84) | 2.00 | 2.00 | 2.00 | 3.00 | 3.00 | 3.00 | 3.00 | 3.00 | 3.00 | <.001 | -0.12 to 0.14 | 0.68 | 0.66 |
| Q5 | 3.24 (0.82) | 3.48 (0.61) | 3.36 (0.73) | 3.00 | 3.00 | 3.00 | 3.00 | 4.00 | 3.00 | 4.00 | 4.00 | 4.00 | <.001 | -0.21 to 0.04 | 0.82 | 0.94 |
| Q6 | 3.14 (0.91) | 3.34 (0.75) | 3.24 (0.84) | 3.00 | 3.00 | 3.00 | 3.00 | 3.00 | 3.00 | 4.00 | 4.00 | 4.00 | <.001 | -0.23 to 0.02 | 0.76 | 0.89 |
| Q7 | 2.79 (0.91) | 3.06 (0.79) | 2.92 (0.86) | 2.00 | 2.50 | 2.00 | 3.00 | 3.00 | 3.00 | 3.50 | 4.00 | 4.00 | <.001 | -0.27 to 0.01 | 0.61 | 0.75 |
| Q8 | 3.37 (0.74) | 3.18 (0.76) | 3.27 (0.75) | 3.00 | 3.00 | 3.00 | 4.00 | 3.00 | 3.00 | 4.00 | 4.00 | 4.00 | .008 | -0.10 to 0.10 | 0.85 | 0.85 |
| Q9 | 3.01 (0.90) | 3.04 (0.84) | 3.03 (0.87) | 2.50 | 2.00 | 2.00 | 3.00 | 3.00 | 3.00 | 4.00 | 4.00 | 4.00 | <.001 | -0.11 to 0.14 | 0.75 | 0.73 |
| Q10 | 3.23 (0.85) | 3.25 (0.81) | 3.24 (0.82) | 3.00 | 3.00 | 3.00 | 3.00 | 3.00 | 3.00 | 4.00 | 4.00 | 4.00 | <.001 | -0.07 to 0.15 | 0.82 | 0.77 |
| Q11 | 2.18 (1.13) | 2.56 (1.05) | 2.37 (1.10) | 1.00 | 2.00 | 1.00 | 2.00 | 2.00 | 2.00 | 3.00 | 3.50 | 3.00 | <.001 | -0.25 to 0.02 | 0.37 | 0.48 |
| Q12 | 2.96 (0.76) | 2.80 (0.79) | 2.88 (0.78) | 2.50 | 2.00 | 2.00 | 3.00 | 3.00 | 3.00 | 3.00 | 3.00 | 3.00 | .008 | -0.01 to 0.24 | 0.75 | 0.63 |
| Q13 | 2.38 (1.11) | 2.72 (1.14) | 2.55 (1.13) | 1.00 | 2.00 | 1.00 | 3.00 | 3.00 | 3.00 | 3.00 | 4.00 | 3.75 | < .001 | -0.24 to 0.04 | 0.51 | 0.61 |
| Q14 | 2.66 (1.09) | 2.70 (1.01) | 2.68 (1.05) | 2.00 | 2.00 | 2.00 | 3.00 | 3.00 | 3.00 | 4.00 | 4.00 | 4.00 | .003 | -0.10 to 0.18 | 0.58 | 0.54 |
| Q15 | 2.37 (1.00) | 2.44 (0.98) | 2.40 (0.99) | 2.00 | 2.00 | 2.00 | 2.00 | 2.00 | 2.00 | 3.00 | 3.00 | 3.00 | <.001 | -0.18 to 0.10 | 0.44 | 0.48 |
| Q16 | 2.46 (0.98) | 2.35 (0.99) | 2.41 (0.98) | 2.00 | 2.00 | 2.00 | 2.00 | 2.00 | 2.00 | 3.00 | 3.00 | 3.00 | .02 | -0.09 to 0.18 | 0.45 | 0.41 |
| Q17 | 2.65 (0.96) | 2.90 (0.99) | 2.77 (0.98) | 2.00 | 2.00 | 2.00 | 3.00 | 3.00 | 3.00 | 3.00 | 4.00 | 4.00 | <.001 | -0.23 to 0.04 | 0.56 | 0.66 |
| Q18 | 2.59 (0.98) | 2.82 (1.03) | 2.70 (1.01) | 2.00 | 2.00 | 2.00 | 3.00 | 3.00 | 3.00 | 3.00 | 4.00 | 3.00 | <.001 | -0.26 to 0.01 | 0.55 | 0.68 |
| Q19 | 2.92 (1.04) | 2.86 (0.98) | 2.89 (1.00) | 2.00 | 2.00 | 2.00 | 3.00 | 3.00 | 3.00 | 4.00 | 4.00 | 4.00 | .007 | -0.16 to 0.10 | 0.65 | 0.68 |
| Q20 | 3.06 (0.86) | 3.18 (0.68) | 3.12 (0.78) | 3.00 | 3.00 | 3.00 | 3.00 | 3.00 | 3.00 | 4.00 | 4.00 | 4.00 | <.001 | -0.18 to 0.04 | 0.77 | 0.85 |
| Q21 | 3.27 (0.76) | 3.37 (0.74) | 3.32 (0.75) | 3.00 | 3.00 | 3.00 | 3.00 | 4.00 | 3.00 | 4.00 | 4.00 | 4.00 | <.001 | -0.13 to 0.08 | 0.82 | 0.85 |
